# Supplementary material for: Automated Identification of Cutaneous Leishmaniasis Lesions Using Deep-Learning-Based Artificial Intelligence
Source: Biomedicines. 2023 Dec 20;12(1):12. doi: 10.3390/biomedicines12010012 (PMC10813291; doi:10.3390/biomedicines12010012)
Supplement: Supplementary file 1 [file biomedicines-12-00012-s001.zip › biomedicines-2716646-supplementary.pdf]

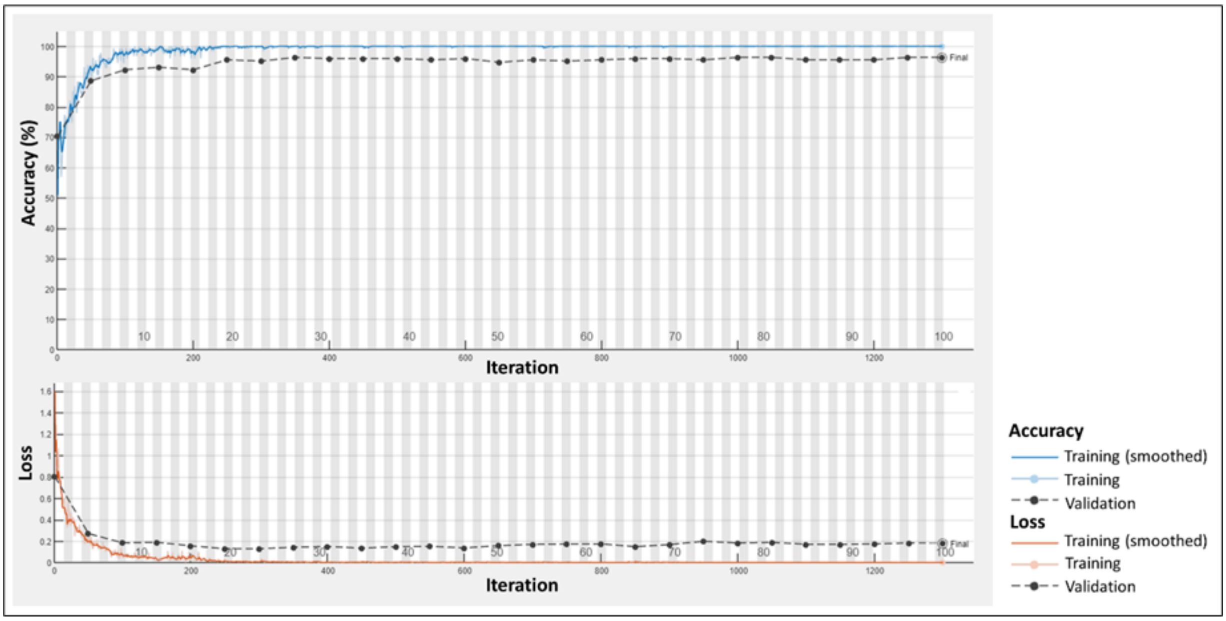

Figure S1. AlexNet network training progress. Curves for training accuracy, validation accuracy, training loss, and validation loss are presented, with smoothing applied to highlight general trends over the course of iterations. The horizontal axis represents the iterations, while the vertical axis represents the corresponding values of the metrics.
